# A peptide derived from adaptor protein STAP-2 inhibits tumor progression by downregulating epidermal growth factor receptor signaling

**DOI:** 10.1016/j.jbc.2022.102724

**Published:** 2022-11-19

**Authors:** Taiga Maemoto, Yuichi Kitai, Runa Takahashi, Haruka Shoji, Shunsuke Yamada, Shiho Takei, Daiki Ito, Ryuta Muromoto, Jun-ichi Kashiwakura, Haruka Handa, Ari Hashimoto, Shigeru Hashimoto, Toyoyuki Ose, Kenji Oritani, Tadashi Matsuda

**Affiliations:** 1 Department of Immunology, Graduate School of Pharmaceutical Sciences, Hokkaido University, Sapporo, Hokkaido, Japan; 2 Faculty of Advanced Life Science, Hokkaido University, Sapporo, Hokkaido, Japan; 3 Department of Molecular Biology, Faculty of Medicine, Hokkaido University, Sapporo, Hokkaido, Japan; 4 Division of Molecular Psychoimmunology, Graduate School of Medicine, Hokkaido University, Sapporo, Hokkaido, Japan; 5 Department of Hematology, International University of Health and Welfare, Narita, Chiba, Japan

**Keywords:** antitumor peptide, prostate cancer, STAT3, EGFR, adaptor protein, DMEM, Dulbecco's modified Eagle's medium, EGFR, epidermal growth factor receptor, FBS, fetal bovine serum, PH, Pleckstrin homology

## Abstract

Signal-transducing adaptor family member-2 (STAP-2) is an adaptor protein that regulates various intracellular signals. We previously demonstrated that STAP-2 binds to epidermal growth factor receptor (EGFR) and facilitates its stability and activation of EGFR signaling in prostate cancer cells. Inhibition of this interaction may be a promising direction for cancer treatment. Here, we found that 2D5 peptide, a STAP-2–derived peptide, blocked STAP-2–EGFR interactions and suppressed EGFR-mediated proliferation in several cancer cell lines. 2D5 peptide inhibited tumor growth of human prostate cancer cell line DU145 and human lung cancer cell line A549 in murine xenograft models. Additionally, we determined that EGFR signaling and its stability were decreased by 2D5 peptide treatment during EGF stimulation. In conclusion, our study shows that 2D5 peptide is a novel anticancer peptide that inhibits STAP-2–mediated activation of EGFR signaling and suppresses prostate and lung cancer progression.

Peptide therapeutics have been developed for the treatment of infectious diseases and cancer because of their biological characteristics such as high selectivity for target molecules and low immunogenicity. Additionally, peptide properties are easily improved by modifications (1, 2, 3, 4, 5). For example, circular peptides have higher stability in blood than linear peptides, and arginine-rich amino acid sequences enhance cellular permeability (1, 6). Moreover, peptides are superior to small compounds for direct and selective blocking of protein–protein interactions because of their high molecular weight. A recent study showed that a blocking peptide for Programmed cell death protein 1 (PD-1)–PD-L1 interactions activates antitumor immune responses and inhibits tumor progression (2).

Epidermal growth factor receptor (EGFR) is a 180 kDa tyrosine kinase receptor activated by its binding to EGF, TGFα, and HB-EGF. Its ligand ligation induces dimerization and phosphorylation of EGFR, resulting in the activation of PI3K/Akt-, STAT3-, and ERK-dependent intracellular signaling (7). Activated EGFR is then ubiquitinated and translocates into endosomes/lysosomes where deubiquitinated EGFR returns to the cell surface, while ubiquitinated EGFR degrades (8, 9). EGFR is necessary for optimal organ development and cell proliferation and correlates with cancer progression and sensitivity for anticancer drugs, including gefitinib, which is an EGFR-targeted tyrosine kinase inhibitor (10, 11). Thus, EGFR is an attractive target for peptide therapeutics against cancers.

Signal-transducing adaptor family member-2 (STAP-2) is an adaptor protein that correlates to various intracellular signals including FAS, MyD88, and FcεRI (12, 13, 14). STAP-2 enhances EGFR-mediated proliferation of DU145 human prostate cancer cells (15). STAP-2 knockdown in DU145 cells decreases their tumor formation in murine xenograft models. Moreover, STAP-2 promotes the proliferation and metastasis of various cancer cell lines such as melanoma, breast cancer, and chronic myeloid leukemia (16, 17, 18). We have also reported that STAP-2 interacts with EGFR *via* its Pleckstrin homology (PH) domain and promotes EGFR stabilization by inhibiting EGFR ubiquitination and degradation in lysosomes (15). Similar to PD-1/PD-L1, development of optimized peptides to inhibit the interactions between STAP-2 and EGFR is likely to have applications in the treatment of EGFR-related malignancies such as lung and prostate cancers.

Here, we found that a STAP-2 PH domain–derived peptide inhibited STAP-2–EGFR interaction and suppressed EGFR-mediated proliferation in several cancer cell lines. The optimized peptide 2D5 inhibited DU145 and A549 tumor growth similarly to STAP-2 knockdown. EGFR signaling and its stability were clearly decreased by 2D5 peptide treatment. These data indicate that our designed peptide may be a novel anticancer drug that inhibits STAP-2–mediated activation of EGFR signaling.

## Results

### STAP-2 PH domain–derived peptides inhibit cancer cell proliferation

**Figure 1:**
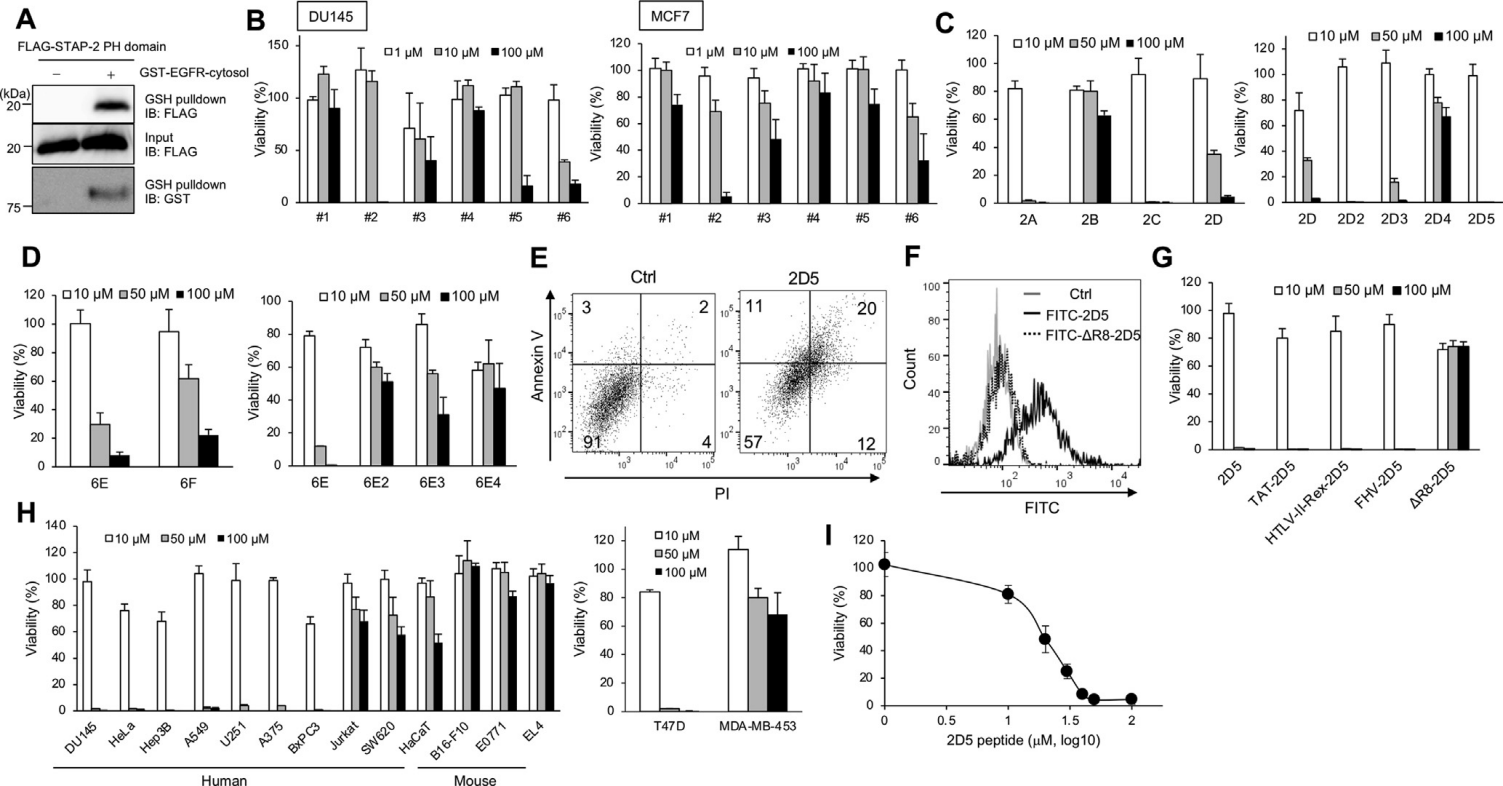
**STAP-2–derived 2D5 peptide inhibits cancer cell proliferation.***A*, HEK293T cells were transfected with expression vectors for the FLAG-STAP-2 PH domain and GST-EGFR cytosol domain, and then lysates were pulled down using glutathione beads at 48 h posttransfection and blotted. *B*–*D*, DU145 cells and MCF7 cells were treated with STAP-2–derived peptides at the indicated concentrations for 48 h, and then relative cell viability was measured by Cell Titer glo assay. *E*, DU145 cells were treated with 50 μM 2D5 peptide for 72 h. Percentage of the cells that were Annexin V and/or PI positive was analyzed by flow cytometry. *F*, DU145 cells were treated with 0.3 μM FITC-conjugated 2D5 and ΔR8-2D5 peptides for 30 min, and then FITC-positive DU145 cells were analyzed by flow cytometry. *G*, DU145 cells were treated with truncated 2D5 peptides, and then cell viability was measured as described in (*B*). *H*, various cell lines were treated with 2D5 peptide, and then cell viability was measured as described in (*B*). T47D cells and MDA-MB-453 cells were treated with 2D5 peptide for 96 h, and then cell viability was measured. *I*, DU145 cells were treated with 1, 10, 20, 30, 40, and 50 μM 2D5 peptide for 48 h, and then relative cell viability was measured by Cell Titer glo assay. n = 3. Mean values and SDs are shown. EGFR, epidermal growth factor receptor; PH, Pleckstrin homology; PI, propidium iodide.

Our previous study demonstrated that STAP-2 interacts with EGFR *via* its PH domain and facilitates EGFR stabilization, implying that STAP-2 PH domain–derived peptides competitively inhibit STAP-2–EGFR interactions and decrease EGFR stability (Fig. 1*A*) (15). We selected six peptide sequences in the human STAP-2 PH domain. Because octa-arginine sequences enhance the cell-penetrating capability of peptides, we conjugated R8 (RRRRRRRRGG) to the N-terminal of the six peptide sequences that are referred as #1 to #6 in Table 1. We investigated whether #1 to #6 peptides inhibited the proliferation of DU145 cells that express both STAP-2 and EGFR. As shown in Figure 1*B*, #2 and #6, but not #1, #3, or #4 peptides suppressed DU145 and MCF7 cell growth. Since #5 peptide had low water solubility and did not suppress MCF7 cell growth, we focused on #2 and #6 sequences and redesigned two series of peptides by shortening the amino acids sequences derived from #2 (#2A–#2D) and #6 (#6E and #6F) peptides. We synthesized the peptide between 52 and 78 residues, but this peptide has very poor solubility and makes large aggregates in the medium. Thus, we did not focus on 57 to 78 residue region in STAP-2 PH domain. #2-derived 2A, 2C, and 2D peptides suppressed DU145 cell growth dose-dependently (Fig. 1*C*, left column). Most amino acid sequences of 2A, 2C, and 2D peptides overlapped (Table 1). Because the 2D peptide was common among the three sequences and the shortest, we further redesigned 2D-derived 2D2, 2D3, 2D4, and 2D5 peptides (Table 1). 2D2, 2D3, and 2D5 peptides, but not the 2D4 peptide, significantly suppressed DU145 cell growth (Fig. 1*C*, right column). As shown in Table 1, 2D2, 2D3, and 2D5 peptide sequences were very similar, and the 2D5 peptide sequence was common among the three sequences and the shortest. Thus, we identify seven amino acids (2D5; GLTIYFY) in the N-terminal of the human STAP-2 PH domain as a possible inhibitory peptide sequence. #6-derived 6E and 6F peptides suppressed DU145 cell growth (Fig. 1*D*, left column). However, their overlapped sequence, #6E4, as well as #6E2 or #6E3 did not inhibit DU145 cell growth (Fig. 1*D*, right column).

**Table 1:** A list of the STAP-2–derived peptides

Peptide	Sequence
#1(P18-K37)	RRRRRRRRGGPSHYYESFLEKKGPCDRDYK
#2(D33-N52)	RRRRRRRRGGDRDYKKFWAGLQGLTIYFYN
#3(T123-E142)	RRRRRRRRGGTDLTLLPGHLYMMSEVLAKE
#4(G78-K97)	RRRRRRRRGGGSSRDPGTHFSLILRDQEIK
#5(D93-F112)	RRRRRRRRGGDQEIKFKVETLECREMWKGF
#6(M108-L127)	RRRRRRRRGGMWKGFILTVVELRVPTDLTL
2A(F39-Q58)	RRRRRRRRGGFWAGLQGLTIYFYNSNRDFQ
2B(F50-N64)	RRRRRRRRGGFYNSNRDFQHVEKLN
2C(Q44-Q58)	RRRRRRRRGGQGLTIYFYNSNRDFQ
2D(Q44-R55)	RRRRRRRRGGQGLTIYFYNSNR
6E(V116-M135)	RRRRRRRRGGVVELRVPTDLTLLPGHLYMM
6F (I113-P122)	RRRRRRRRGGILTVVELRVP
2D2(Q44-Y51)	RRRRRRRRGGQGLTIYFY
2D3(G45-N52)	RRRRRRRRGGGLTIYFYN
2D4(Q44-Y49)	RRRRRRRRGGQGLTIY
2D5(G45-Y51)	RRRRRRRRGGGLTIYFY
6E2(V121-G130)	RRRRRRRRGGVPTDLTLLPG
6E3(V116-G130)	RRRRRRRRGGVVELRVPTDLTLLPG
6E4(V116-P122)	RRRRRRRRGGVVELRVP
TAT-2D5	GRKKRRQRRRPPQGGLTIYFY
HTLV-II-Rex-2D5	TRRQRTRRARRNRGGLTIYFY
FHV-2D5	RRRRNRTRRNRRRVRGGLTIYFY
ΔR8-2D5	GLTIYFY

We focused on 2D5 peptide to clarify further effects. 2D5 peptide induced cell death in DU145 cells (Fig. 1*E*). Incorporation of 2D5 peptide into DU145 cells was dependent on the R8 sequence (Fig. 1*F*). We next designed 2D5-derived peptides by converting the R8 sequence in 2D5 peptide to other cell-penetrating peptides, which were derived from HIV TAT (GRKKRRQRRRPPQGG), HTLV-II Rex (TRRQRTRRARRNRGG), and FHV (RRRRNRTRRNRRRVRGG). These 2D5-derived peptides, but not 2D5 peptide without the R8 sequence, suppressed DU145 cell growth, indicating that the cytotoxic effects of 2D5 peptide required a cell-penetrating capability (Fig. 1*G*). 2D5 peptide inhibited the growth of various human EGFR-expressing cell lines, but not in human T cell line Jurkat or human keratinocyte-like cell line HaCaT, suggesting that 2D5 peptide was less cytotoxic in MDA-MB-453 cells and SW620 cells, EGFR-negative human cell lines, and noncancer cells like HaCaT cells (Fig. 1*H*). Additionally, 2D5 peptide did not inhibit the proliferation of murine cancer cell lines such as B16-F10, E0771, and EL4 (Fig. 1*H*). We assessed the inhibitory effect of 2D5 peptide on DU145 cell growth at various concentrations and its IC_50_ was calculated to be 19.4 μM from Cell Titer glo assay (Fig. 1*I*). These data indicated that 2D5 peptide, a human STAP-2 PH domain-derived peptide, with a cell-penetrating capability significantly inhibited cancer cell growth.

### 2D5 peptide suppresses EGFR signaling

**Figure 2:**
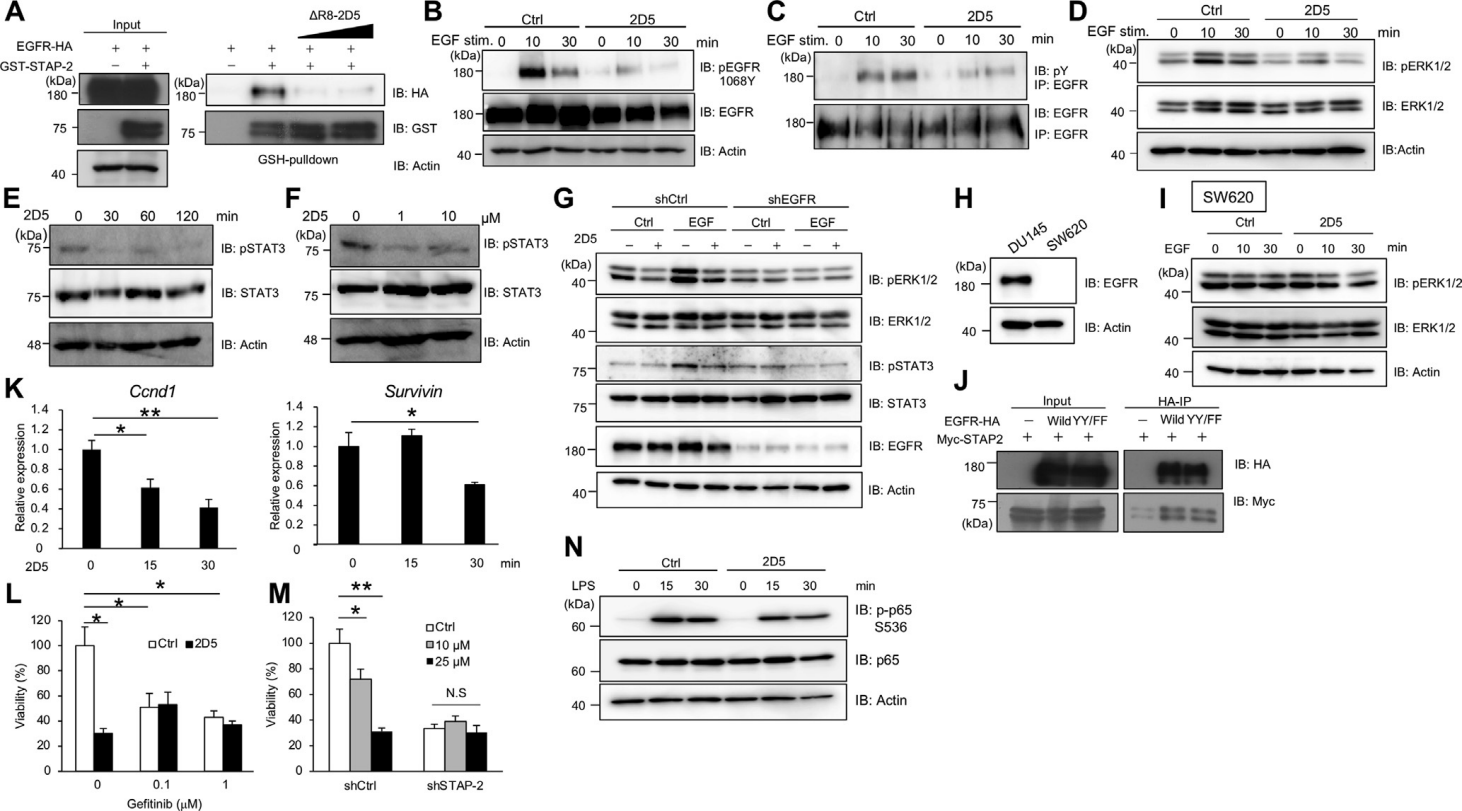
**2D5 peptide suppresses EGFR signaling.***A*, HEK293T cells were transfected with expression vectors for GST-STAP-2 and EGFR-HA, and then lysates were incubated with 1 or 50 μM ΔR8-2D5 peptide for 1 h. GST-STAP-2 was pulled down and blotted. *B*–*D*, DU145 cells were serum starved for 1 h and then treated with 10 μM 2D5 peptide for 30 min. The cells were stimulated with 100 ng/ml EGF for 0, 10, and 30 min and then lysates were blotted. *E*, DU145 cells were treated with 10 μM 2D5 peptide for 0, 30, 60, and 120 min, and then lysates were blotted. *F*, DU145 cells were treated with 1 or 10 μM 2D5 peptide for 30 min, and then lysates were blotted. *G*, shCtrl- and shEGFR-expressing DU145 cells were treated with 2D5 peptide same as (*B*) and stimulated with 100 ng/ml EGF for 15 min, then lysates were blotted. *H* and *I*, SW620 cells were treated with 2D5 peptide and 100 ng/ml EGF same as (*B*), and then lysates were blotted. *J*, HEK293T cells were transfected with expression vectors for Myc-STAP-2 and EGFR-HA WT or Y1068F/Y1173F (YY/FF), and then lysates were immunoprecipitated and blotted. *K*, DU145 cells were treated with 10 μM 2D5 peptide, and then Ccnd1 and Survivin mRNA levels were quantified by qPCR. *L*, DU145 cells were treated with 25 μM 2D5 peptide and 0.1 or 1 μM gefitinib for 48 h, and then cell viability was measured. *M*, shCtrl- and shSTAP-2-expressing DU145 cells were treated with 25 μM 2D5 peptide for 48 h, and then cell viability was measured. *N*, THP-1 cells were treated with 20 ng/ml PMA for 3 days. THP-1–derived macrophages were treated with 10 μM 2D5 peptide for 1 h and then stimulated with 1 μg/ml LPS for the indicated times, and then lysates were blotted. n = 3, Mean values and SDs are shown. ∗*p* < 0.05, ∗∗*p* < 0.01 (paired Student’s *t* test). EGFR, epidermal growth factor receptor; PMA, phorbol 12-myristate 13-acetate; qPCR, quantitative PCR.

Next, we investigated whether 2D5 peptide inhibited STAP-2–EGFR interactions and suppressed EGFR signaling. ΔR8-2D5 peptide, which lacked the octa-arginine sequence, inhibited the *in vitro* interaction between STAP-2 and EGFR (Fig. 2*A*). Tyrosine phosphorylation of EGFR is important for activation of its downstream signaling. Phosphorylation of Y1068 in EGFR is required for EGFR-mediated STAT3 and ERK activation. Conversely, Y1173 phosphorylation of EGFR suppresses ERK activation through SHP-2. 2D5 peptide decreased EGF-induced Y1068 and total tyrosine phosphorylation of EGFR and suppressed phosphorylation of STAT3 and ERK (Fig. 2, *B*–*F*). 2D5 peptide did not decrease the phosphorylation of ERK and STAT3 in EGFR-knockdown DU145 cells and SW620 cells (Fig. 2, *G*–*I*). Y1068 and Y1173 phosphorylation of EGFR was not required for its binding to STAP-2, suggesting that 2D5 peptide may inhibit STAP-2–EGFR interactions independent of Y1068 and Y1173 phosphorylation of EGFR (Fig. 2*J*). 2D5 peptide decreased the mRNA levels of Ccnd1 and Survivin that are target genes of EGFR signaling (Fig. 2*K*). Although both 2D5 peptide and gefitinib inhibited DU145 cell growth, additional or synergistic effects were not observed (Fig. 2*L*). These data indicated that the mechanism of cytotoxicity caused by 2D5 peptide treatment overlapped with gefitinib in EGFR signaling. Cytotoxicity of 2D5 peptide disappeared in STAP-2–knockdown DU145 cells, indicating that 2D5 peptide inhibited cancer cell growth dependently on STAP-2 expression (Fig. 2*M*). Moreover, 2D5 peptide treatment did not affect MyD88-mediated signaling. 2D5 peptide did not inhibit LPS-induced NF-κB activation in THP-1–derived macrophages (Fig. 2*N*). These data indicated that 2D5 peptide inhibited STAP-2–EGFR interactions and decreased cancer cell growth *via* downregulation of EGFR signaling.

### 2D5 peptide facilitates EGFR localization to lysosomes and its degradation after EGF stimulation

**Figure 3:**
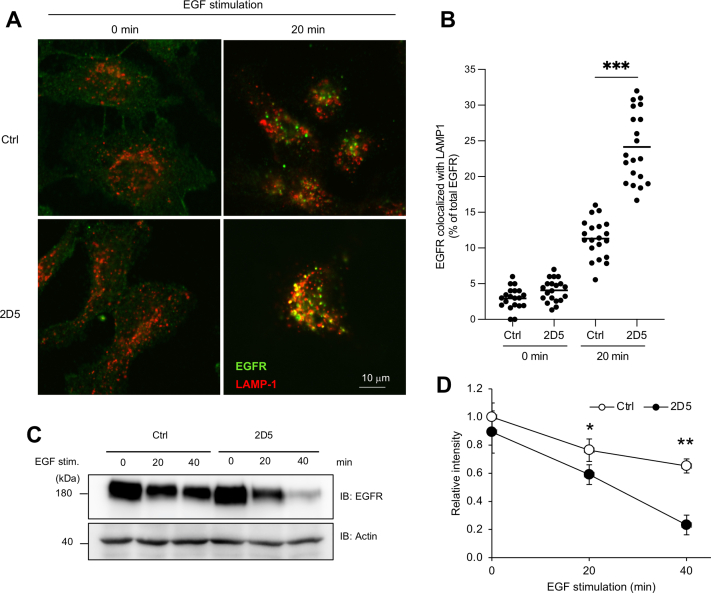
**2D5 peptide decreases EGFR stabilization.***A*, DU145 cells were treated with 10 μM 2D5 peptide for 1 h under serum starvation and then stimulated with 100 ng/ml EGF for 20 min. The cells were fixed and stained with anti-EGFR (*green*) and anti-LAMP1 (*red*) antibodies. *B*, localization of EGFR and LAMP-1 was observed by confocal microscopy. We calculated the area of total EGFR and EGFR-LAMP-1 colocalization by an ImageJ software, and the percentage of EGFR-LAMP-1/total EGFR area in each 20 cells were shown. n = 20. *C*, DU145 cells were treated with 10 μM cycloheximide in serum-free medium for 1 h and then treated with 50 μM 2D5 peptide for 1 h together with 10 μM cycloheximide in serum-free medium. The cells were then stimulated with 100 ng/ml EGF, and lysates were blotted. *D*, band intensity of EGFR/Actin in (*C*) (n = 3). Mean values and SDs are shown. ∗*p* < 0.05, ∗∗*p* < 0.01, ∗∗∗*p* < 0.001 (paired Student’s *t* test). EGFR, epidermal growth factor receptor.

We previously demonstrated that EGFR localization to lysosomes increases in STAP-2–knockdown DU145 cells during EGF stimulation (15). These data indicate that STAP-2 promotes EGFR stabilization by inhibiting EGFR translocation and degradation in lysosomes. Thus, we investigated whether 2D5 peptide enhanced translocation to lysosomes and EGFR degradation. EGFR proteins translocated from the surface membrane to lysosomes in response to EGF stimulation. As expected, this translocation was enhanced by 2D5 peptide treatment (Fig. 3, *A* and *B*). Additionally, EGFR proteins disappeared more rapidly after 2D5 peptide treatment of EGF-stimulated DU145 cells (Fig. 3*C*). After EGF stimulation, the half-life of EGFR proteins was approximately 25 min in 2D5-treated DU145 cells, but more than 40 min in control cells (Fig. 3*D*). These data indicated that 2D5 peptide decreased EGFR stability during EGF stimulation by promoting EGFR translocation to lysosomes.

### Administration of 2D5 peptide inhibits tumor progression in a murine xenograft model.

**Figure 4:**
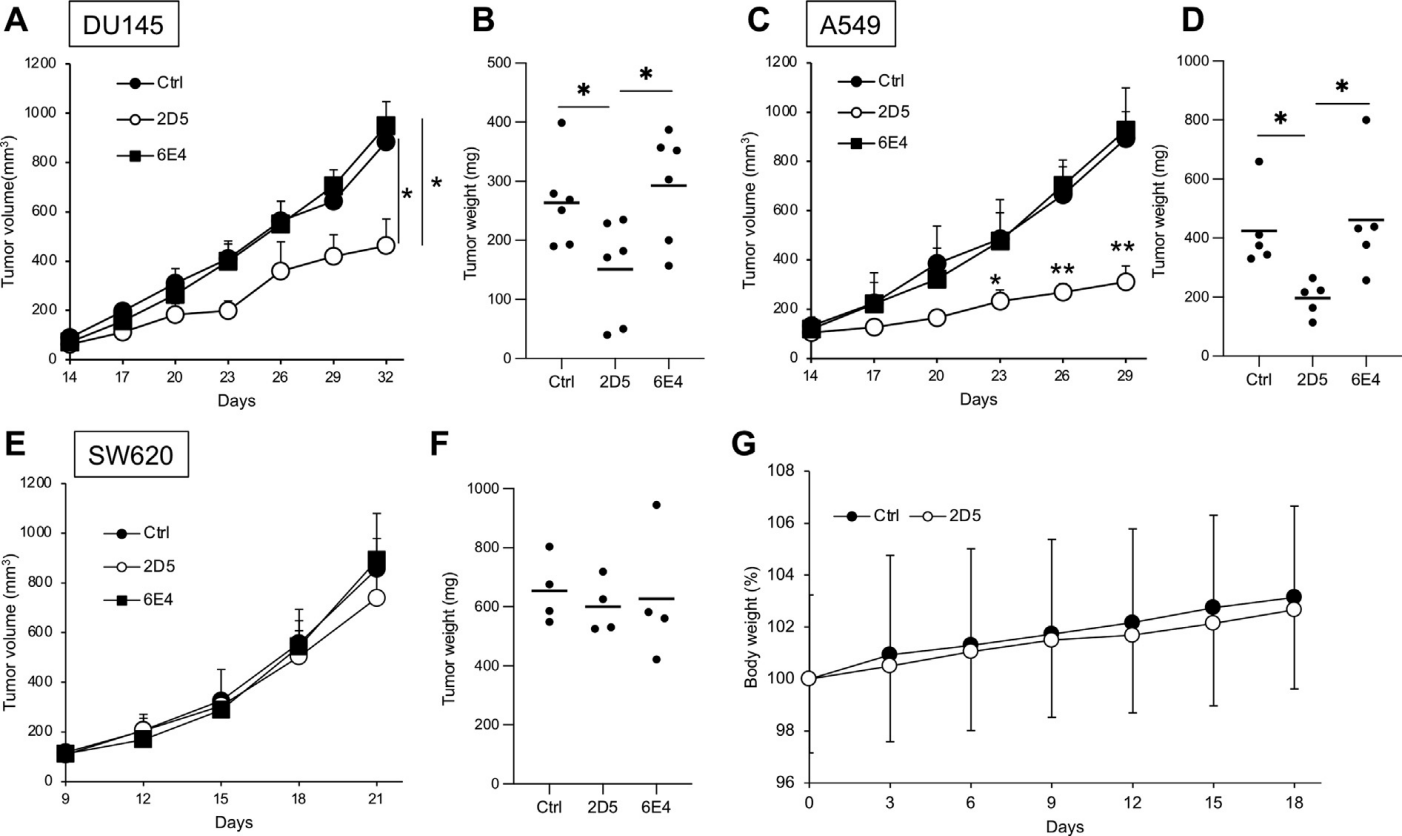
**2D5 peptide inhibits tumor growth in tumor-bearing mice.***A*, DU145 cells were subcutaneously injected into BALB/c-nude mice and then saline, and 2 mg/mouse 6E4 or 2D5 peptides were administered by intratumoral injection at day 14, 17, and 20. Tumor volume at days 14 to 32 and (*B*) tumor weight at day 32 are shown. n = 6. *C*, A549 cells were subcutaneously injected into BALB/c-nude mice, and then peptides were administered as described in (*A*). *D*, A549 tumor weight was measured at day 29. n = 5. *E*, SW620 cells were subcutaneously injected into BALB/c-nude mice, and then peptides were administered at day 9, 12, and 15 same as (*A*). Tumor volume at days 9 to 21 and (*F*) tumor weight at day 21 are shown. n = 4. *G*, BALB/c-nude mice were administered saline or 2 mg/mouse 2D5 peptide by subcutaneous injection at day 0, 3, and 6. Mouse body weights are shown. n = 3. Mean values and SDs are shown. ∗*p* < 0.05 (paired Student’s *t* test).

Finally, we investigated whether 2D5 peptide inhibited tumor growth in tumor-bearing mice. 2D5 peptide injection inhibited tumor formation of DU145 and A549 cells, but a control peptide (6E4), which had shown no cytotoxic effects on cancer cell growth *in vitro*, did not affect tumor development (Figs. 1*D* and 4, *A*–*D*). Furthermore, 2D5 peptide did not show antitumor effects on SW620 tumor in murine xenograft model (Fig. 4, *E* and *F*). Administration of 2D5 peptide did not affect mouse body weight, suggesting that 2D5 peptide has less toxicity *in vivo* (Fig. 4*G*). These data indicated that 2D5 peptide had *in vivo* antitumor effects on EGFR-driven prostate and lung cancer progression.

## Discussion

Adaptor proteins correlate to cancer progression by regulating various intracellular signals induced by cytokines, growth factors, and integrins (19, 20, 21, 22). Thus, adaptor proteins are attractive targets to develop anticancer drugs. However, inhibition of adaptor proteins by small compounds is difficult because of the lack of their enzymatic activity. Peptides bind to target proteins with high specificity and interfere with protein–protein interactions, suggesting that well-designed peptides have the potential to be inhibitors of adaptor proteins.

Our previous studies have shown that STAP-2 binds to various intracellular signaling molecules including EGFR, Fas, BCR-ABL, and MyD88 (12, 13, 15, 18). MyD88 interacts with the SH2 domain of STAP-2 and promotes cytokine production by upregulating IKK-mediated NF-κB activation (13). 2D5 peptide treatment did not affect LPS-induced NF-κB activation in THP-1 macrophages, indicating that 2D5 peptide does not inhibit STAP-2–mediated MyD88 regulation (Fig. 2*N*). Thus, 2D5 peptide selectively inhibits STAP-2 PH, but not SH2, domain functions. This selectivity is likely to be derived from highly specific sequence recognition by the peptide.

2D5 peptide inhibited the proliferation of human, but not murine, cancer cells, suggesting that STAP-2 contributes to EGFR-mediated proliferation of human cancer cells, but not murine cancer cell lines (Fig. 1*H*). The 2D5 peptide region in STAP-2 has 71% identity (five out of seven amino acids), although the cytosolic domain of EGFR has 96% identity between humans and mice. STAP-2 knockdown decreases the proliferation in B16-F10 cells, but we did not find a STAP-2–EGFR complex in a murine cell line (data not shown). Thus, we speculate that murine STAP-2 enhances EGFR-independent cell proliferation and does not affect EGFR signaling in murine cancer cells.

Cytotoxicity in normal cells and tissues is a major problem in the development of antitumor drugs, because it causes unexpected side effects during clinical trials. 2D5 peptide showed antitumor effects against prostate and lung cancer *in vitro* and *in vivo* but did not inhibit the proliferation of noncancer cells (Fig. 1*H*). Furthermore, administration of 2D5 peptide clearly inhibited prostate and lung cancer cell growth but did not decrease body weight of the murine xenograft model (Fig. 4*G*). Additionally, STAP-2–deficient mice have no obvious phenotypes under a steady-state condition (23). These findings suggest that 2D5 peptide has less cytotoxic effects on normal cells and tissues than common EGFR inhibitors such as gefitinib and erlotinib. Numerous studies have shown that EGFR and its downstream signals are overactivated in various cancer cell lines, but not in normal cells, suggesting that 2D5 peptide with high sequence specificity is preferentially cytotoxic against EGFR-overactivated cells (7, 11). In support of this idea, several cancer cells overexpress EGFR-stabilizing proteins, such as Sortilin, TRIB3, and USP22, which suppress EGFR degradation, and their knockdown inhibits cancer cell proliferation (24, 25, 26). Therefore, these “EGFR stabilizers”, including STAP-2, are good targets for the treatment of EGFR-overactivated cancers such as nonsmall cell lung, prostate, and breast cancers.

The IC_50_ of 2D5 peptide in DU145 cells was 19.4 μM *in vitro* (Fig. 1*I*). 2D5 peptide should be improved to inhibit cancer cell growth at lower concentrations because general antitumor drugs are required for antiproliferative effects at the sub-micro molar concentration *in vitro*. In this regard, 2D5 peptide might be improved by increasing its stability and affinity for EGFR. Recent studies have reported that stapled peptides have higher affinity for target molecules, more resistance against proteases, and a higher cell-penetrating capability (27). EGFR is degraded in lysosomes after EGF stimulation, implying that 2D5 peptide is also degraded together with EGFR in lysosomes. Thus, a stapled 2D5 peptide may have higher resistance against lysosomal proteases and anticancer effects *in vitro* and *in vivo* at lower concentrations similarly to general anticancer drugs.

Adaptor proteins have been considered to be undruggable targets for many years but have become druggable targets through the development of new peptide techniques. In this study, we showed that 2D5 peptide acted as an inhibitor of STAP-2–EGFR interactions and suppressed EGFR-mediated cancer cell growth. Thus, 2D5 peptide-inhibitor may be used as a novel antitumor drug in the future.

## Experimental procedures

### Animals

All animals were kept under specific pathogen-free conditions. BALB/c-nude mice were purchased from Sankyo Labo Service Corporation, Inc. Animal experiments were performed with the approval of the Animal Research Committee of Hokkaido University.

### Reagents and cells

Gefitinib was purchased from Tokyo Chemical Industry. Cycloheximide was purchased from Wako. phorbol 12-myristate 13-acetate was purchased from Sigma. Peptides were purchased from GenePharma. Amino acid sequences of peptides were described in Table 1. SW620 and MDA-MB-453 cells were cultured in Leibovitz's L-15 Medium (Wako) supplemented with 10% fetal bovine serum (FBS; Gibco, Thermo Fisher Scientific) at 37 °C in a humidified atmosphere without CO_2_. Other cell lines were cultured in Dulbecco’s modified Eagle’s medium (DMEM; Sigma) supplemented with 10% FBS and 0.05 mM 2-mercaptoethanol (Nacalai Tesque) at 37 °C in a humidified atmosphere with 5% CO_2_.

### Plasmid construction

The construction of STAP-2 expression vectors has been described previously (28). EGFR expression vectors were kind gifts from Dr J. N. Ihle (St Jude Children’s Research Hospital) and Dr H. Sakurai (Toyama University) (29). The EGFR Y1068F/Y1173F expression vector was prepared by site-directed mutagenesis using pCIneo-hEGFR-HA. To construct an expression vector for the GST-EGFR cytosol domain, complementary DNA of the EGFR cytosolic domain (645–1211 aa) was amplified by PCR using pCIneo-hEGFR-HA and inserted into the BamHI/NotI sites in the pEBG vector.

### Cell proliferation assay

DU145 cells (5 × 10^3^/well) were seeded in a 96-well plate, cultured for 24 h, and then treated with STAP-2–derived peptides for the indicated periods. Twenty five microliters of 0.5% NP-40 and 2% Cell Titer Glo 2.0 (Promega) was added to the cells and then the cells were incubated for 10 min at room temperature with gentle shaking. Then, 100 μl of samples were transferred to a well of a 96-well white plate (Greiner Bio-One) and luminescence was measured by an Infinite M200 (TECAN).

### Annexin V/propidium iodide staining

DU145 cells were cultured in 6-well plates and treated with 2D5 peptide for 72 h, and then the cells were collected by trypsinization and stained with 160 ng/ml Annexin V-APC and 1 μg/ml propidium iodide for 15 min on ice. The cells were analyzed by a Gallios flow cytometer (Becton Dickinson).

### Knockdown

Preparation of shRNA-expressing DU145 cells was described as previously (15). pLKO.1-shEGFR was purchased from Sigma (TRCN00000295968).

### Quantitative PCR

Total RNA was purified from DU145 cells using TRI reagent (Thermo Fisher Scientific) in accordance with the manufacturer’s instructions. Complementary DNA was synthesized from the RNA using ReverTra Ace (TOYOBO). mRNA levels of target genes were quantified using KAPA SYBR green mix (KAPA biosystems) with a CFX connect (Bio-Rad). Primer sets for quantitative PCR analysis were as follows: hGAPDH fwd, 5′-gaaatcccatcaccatcttccagg-3′; hGAPDH rev, 5′-cagtagaggcagggatgatgttc-3′; hCyclin D1 fwd, 5′-aactacctggaccgcttcct-3′; hCyclin D1 rev, 5′-ccacttgagcttgttcacca-3′; hSurvivin fwd, 5′-ggaccaccgcatctctacat-3′; hSurvivin rev, 5′-gacagaaaggaaagcgcaac-3′.

### Immunoblotting

DU145 cells were treated with 2D5 peptide at the indicated concentrations and for the indicated periods and then lysed in lysis buffer (20 mM Tris–HCl, pH 8.0, 150 mM NaCl, 1 mM EDTA, 1% NP-40, 0.5% deoxycholate, and 0.1% SDS) with sonication. After centrifugation, the supernatants were subjected to SDS-PAGE and then immunoblotted. The following antibodies were used for immunoblotting: anti-Myc antibody (9E10, Sigma), anti-HA antibody (C29F4, Cell Signaling Technology), anti-EGFR antibody (D38B1, Cell Signaling Technology), anti-phospho-EGFR Y1068 antibody (Cell Signaling Technology), anti-GST antibody (Z-5, Santa Cruz Biotechnology), anti-STAT3 antibody (79D7, Cell Signaling Technology), anti-phospho-STAT3 antibody (GeneTex), anti-ERK antibody (K-23, Santa Cruz Biotechnology), anti-phospho-ERK antibody (Cell Signaling Technology), anti-β-actin antibody (AC-15, Santa Cruz Biotechnology), anti-p65 antibody (D14E12, Cell Signaling Technology), anti-phospho-p65 antibody (93H1, Cell Signaling Technology), and anti-phosphotyrosine antibody (4G10, GeneTex).

### Immunoprecipitation

HEK293T cells were transfected with expression vectors for Myc-STAP-2 and EGFR-HA using PEI MAX (MW: 40,000, Polysciences) in accordance with the manufacturer’s instructions and then lysed in lysis buffer at 48 h posttransfection. EGFR-HA in the lysate was immunoprecipitated using an anti-HA antibody (F-7, Santa Cruz Biotechnology) and blotted.

### Pull-down assay

HEK293T cells were transfected with expression vectors for GST-STAP-2 and EGFR-HA using PEI MAX in accordance with manufacturer’s instructions and then lysed in 20 mM Tris–HCl, pH 8.0, 150 mM NaCl, 1 mM EDTA, and 1% NP-40. The lysates were incubated with 2D5 peptide for 1 h at 4 °C and then GST-STAP-2 was pulled down for 90 min using glutathione beads. After washing, GST-STAP-2 was eluted in 0.1 M Tris–HCl, pH 9.0, and 150 mM glutathione for 15 min with vigorous shaking. Eluted proteins were analyzed by immunoblotting.

### Immunofluorescence

DU145 cells were grown on 12-mm glass coverslips (Matsunami Glass Industry) in DMEM containing 10% FBS. The cells were cultured for 1 h in serum-free DMEM containing 2D5 peptide for 1 h and then stimulated with 100 ng/ml human recombinant EGF (PeproTech) for 20 min. The cells were fixed with cold methanol for 30 min and then LAMP-1 and EGFR were stained as described previously (15). Images were obtained by confocal microscopy under an FV-10 microscope (Olympus).

### Administration of 2D5 peptide to tumor-bearing mice

BALB/c-nude mice (female, 4 weeks old) were subcutaneously injected with 6 × 10^6^ DU145 cells, 3 × 10^6^ A549 cells, or 2 × 10^6^ SW620 cells with Matrigel (Corning Inc). 2D5 peptide (2 mg/mouse) was injected into the tumor-bearing mice at days 14, 17, and 20 *via* intratumoral injection.

## Data availability

The datasets generated and/or analyzed during the current study are available from the corresponding author on reasonable request.

## Conflict of interest

All authors declare no conflicts of interest in regard to this article.
